# 3D-Printed Immunosensor Arrays for Cancer Diagnostics

**DOI:** 10.3390/s20164514

**Published:** 2020-08-12

**Authors:** Mohamed Sharafeldin, Karteek Kadimisetty, Ketki S. Bhalerao, Tianqi Chen, James F. Rusling

**Affiliations:** 1 Department of Chemistry, University of Connecticut, Storrs, CT 06269, USA; mohamed.sharafeldin@uconn.edu (M.S.); ketki.bhalerao@uconn.edu (K.S.B.); tianqi.chen@uconn.edu (T.C.); 2 LifeSensors Inc., 271 Great Valley Parkway, Suite 100, Malvern, PA 19355, USA; karteek.kadimisetty@gmail.com; 3 Department of Surgery and Neag Cancer Center, UConn Health, Farmington, CT 06032, USA; 4 School of Chemistry, National University of Ireland at Galway, Galway H91 TK33, Ireland

**Keywords:** 3D printing, POC, microfluidics, immunosensor, cancer, biomarkers

## Abstract

Detecting cancer at an early stage of disease progression promises better treatment outcomes and longer lifespans for cancer survivors. Research has been directed towards the development of accessible and highly sensitive cancer diagnostic tools, many of which rely on protein biomarkers and biomarker panels which are overexpressed in body fluids and associated with different types of cancer. Protein biomarker detection for point-of-care (POC) use requires the development of sensitive, noninvasive liquid biopsy cancer diagnostics that overcome the limitations and low sensitivities associated with current dependence upon imaging and invasive biopsies. Among many endeavors to produce user-friendly, semi-automated, and sensitive protein biomarker sensors, 3D printing is rapidly becoming an important contemporary tool for achieving these goals. Supported by the widely available selection of affordable desktop 3D printers and diverse printing options, 3D printing is becoming a standard tool for developing low-cost immunosensors that can also be used to make final commercial products. In the last few years, 3D printing platforms have been used to produce complex sensor devices with high resolution, tailored towards researchers’ and clinicians’ needs and limited only by their imagination. Unlike traditional subtractive manufacturing, 3D printing, also known as *additive manufacturing*, has drastically reduced the time of sensor and sensor array development while offering excellent sensitivity at a fraction of the cost of conventional technologies such as photolithography. In this review, we offer a comprehensive description of 3D printing techniques commonly used to develop immunosensors, arrays, and microfluidic arrays. In addition, recent applications utilizing 3D printing in immunosensors integrated with different signal transduction strategies are described. These applications include electrochemical, chemiluminescent (CL), and electrochemiluminescent (ECL) 3D-printed immunosensors. Finally, we discuss current challenges and limitations associated with available 3D printing technology and future directions of this field.

## 1. Introduction

Cancer is one of the leading causes of death worldwide. Globally, it was responsible for approximately 9.6 million deaths in 2018 [1]. A major contributing factor to the high mortality is late diagnosis due to the unavailability of modern diagnostic tools in low income countries and their limited accessibility or application in developed countries. Currently, cancer diagnosis rely on techniques such as magnetic resonance imaging (MRI), computed tomography (CT), endoscopy, mammography and pathological examination of tissue biopsies [2,3,4]. Because the tumor needs to be located first with these techniques, in the majority of cancer cases, cancers will only be found as patients start to show symptoms, where treatment options become limited and health is already in jeopardy [5]. Providing early diagnosis and effective screening for different cancers are major challenges to improve life expectancy and treatment outcomes [6].

**Figure 1:**
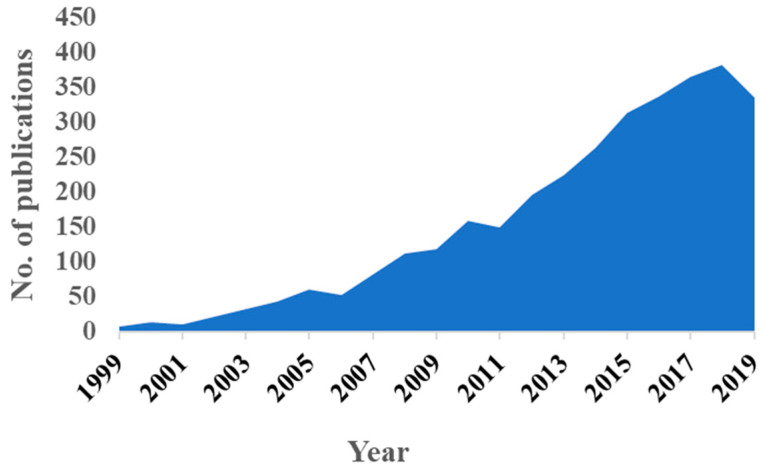
Number of publications per year focusing on protein biomarker cancer diagnostics from 1999–2019. Results generated using web of science^®^ report generation tool for “Cancer Protein sensors” on 9 May 2020.

The crucial need for effective cancer screening and accessible diagnostic tools has driven research endeavors utilizing cancer biomarkers in liquid biopsy samples like blood, urine, and saliva. Analyzing cancer markers in liquid biopsy samples overcome hurdles associated with solid tumor biopsy as it provides a rapid, precise, and non-invasive assay strategy [7,8], and does not require a tumor to be located. Protein biomarkers provide an opportunity to assess risk of cancer development and to detect cancer at very early stage where treatment interventions are most effective [9]. Sensors utilizing ligand-binding assay formats for candidate cancer protein biomarkers have drawn a remarkable interest in the last two decades indicated by increased number of publications as seen in Figure 1.

Several analytical strategies have been adapted for development of ultrasensitive detection of protein biomarkers associated with different types of cancer. Immunoassay format is the most commonly used technique for analysis due to the inherent specificity associated with the use of antibodies as molecular-recognition agents [10,11]. Immunoassay formats have been integrated with several detection strategies in order to develop cancer diagnostics including colorimetric [12], fluorescence [13], electrochemical [14], chemiluminescence [15], electrochemiluminescence [16], and plasmon resonance sensors [17].

The vast development of sensor assembly techniques encompassed a great leap in the progress of immunoassay-based cancer biomarker diagnostics. Several immunoassay-based diagnostic tools have been recently commercialized with promises of unprecedented sensitivities including electrochemiluminescence-based Meso Scale Discovery (MSD) platform and single molecule array technology (Simoa^®^ technology) by Quanterix^®^ (MA, USA) [18,19]. Although these techniques provided an excellent opportunity for early diagnosis and understanding cancer biology, they are limited to centralized laboratories as they require expensive bulky instrumentation and trained operators. With advanced manufacturing techniques, sensors developed acquired better automation, higher sensitivities, far-reaching accessibility, and multiplexing capabilities [20,21]. These developments promise the realization of point-of-care (POC) testing for cancer screening, detection, and staging. Among various approaches utilized for fulfilling these POC testing requirements, additive manufacturing furnished a launchpad for innovative yet easy cancer biomarker sensor manufacturing tool [22].

Additive manufacturing, also known as 3D printing, is making rapid inroads in manufacturing, and advanced fabrications that are quickly moving into production [23]. 3D printing has been utilized in development and fabrication of sensors for detection of glucose [24], drugs [25], trace elements [26], neurotransmitters [27], nucleic acids [28], and proteins [29]. The vast scope and innovative nature of 3D printing in development of biosensors is backed by the versatility of options provided by the immense progress in the design and production of desktop 3D printers. These 3D printers now offer access to hundreds of printable substrate materials that can be used to make products with spectrum of properties including transparency, electrical conductivity, elasticity, chemical and thermal resistivity. This has allowed the design and fabrication of previously hard to rapidly fabricate sensors and sensor arrays at very low cost.

The process of 3D printing is rather simple, a computer software often available at no charge to academics, is used to create the initial design. The initial computer aided design (CAD) file is then sliced into printable layers using slicing software specific for each desktop 3D printer. The 3D printer then physically prints layers on top of each other to form the final product [30]. Recently, a more advanced printing technique, tomographic volumetric 3D printing, has been used to print the whole design in one step eliminating the need for slicing and layer-by-layer printing which in turn drastically reduces printing time [31].

In this review, we provide a summary of different 3D printing techniques currently utilized in desktop 3D printers. In addition, we describe, as examples, the application of the 3D printing technology for development and fabrication of electrochemical, chemiluminescence, and electrochemiluminescence sensors for cancer biomarker proteins. We also discuss the design of complex hybrid sensors that can be achieved with 3D printing. Finally, we give a brief account on limitations associated with current 3D printing technologies and possible future impact of 3D printing.

## 2. 3D Printing Technologies

Several strategies have been adapted in the production of desktop 3D printers depending on the principle of printing and nature of printable substrate. Printable substrates can be divided into polymerizable materials, thermoplastics, and curable inks. Based on the nature of the substrate material different printing technologies have been developed. In this section, we will describe common 3D printing technologies, principles, limitations, and applications.

### 2.1. Fused Deposition Modeling (FDM)

**Figure 2:**
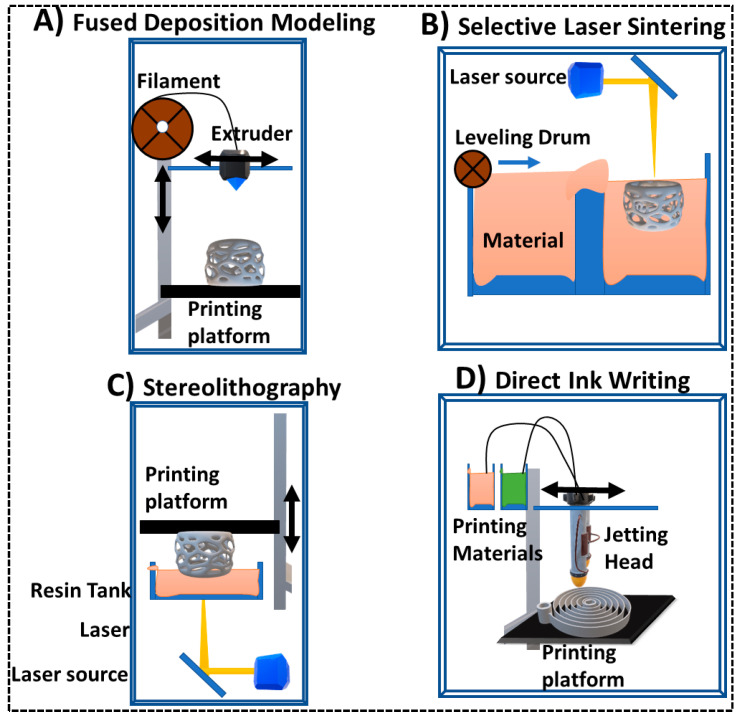
Schematic illustration of 3D printing techniques commonly utilized in prototyping and production of cancer immunosensors. Arrows indicates direction of movement.

FDM is one of the first 3D printing techniques utilized in desktop 3D printers commercially available at a large scale. This method utilizes a thermoplastic material which is melted through a heated printing nozzle and extruded onto solid printing platform. The printing nozzle head moves in X, Y, and Z directions in order to extrude layers on top of each other. Once extruded out of the printing head, thermoplastics tend to restore their solid nature before being heated in the printing nozzle (Figure 2A). FDM offers a low-cost 3D printing technology with easily changeable materials and minimal waste [32]. It also allows printing of multi-component parts simultaneously with printers equipped with double or triple printing nozzles [33]. The most common substrate materials utilized in FDM printers are acrylonitrile butadiene styrene (ABS) and polylactic acid (PLA) [34]. Interestingly, FDM was successfully utilized in printing of carbonaceous conductive substrates like carbon black, graphene, or carbon nanotubes mixed with thermoplastic materials [35]. Printing conductive materials paved the way for 3D printing of electronic components [36], integrated electrochemical sensors [37], and batteries [38]. FDM suffers from several drawbacks associated with low printing resolution (~5 µm), relatively high energy requirements, hazardous vapors, and adhesion problems with multi-materials printing [39].

### 2.2. Photopolymerization/Stereolithography (SLA)

Photopolymerization and digital light processing utilizes a selective light-aided curing process of special polymerizable liquid where polymerization is induced by light of specific wavelength. A moving laser beam hitting a resin-filled vat at a programmed pattern controlled by the printer software induces polymerization at that specific point. As laser beam moves, it cures a pre-designed layer point-by-point onto a solid printing platform that was immersed into the resin tank at a very close proximity to the tank bottom. After each layer the printing platform moves in the Y direction for next layer to be printed allow printing of successive layers on top of each other (Figure 2C). Digital light processing utilizes a similar system while curing one layer at each projection which allow faster printing compared to point-by-point curing [40]. Polymerizable liquids usually consist of monomers and oligomers of epoxides and acrylates mixed with photo-initiators. Light focused onto a single point or a projected layer activates crosslinking of the monomers and/or oligomers in the liquid mixture into a solid polymer. The moving printing platform allows the liquid mixture to fill the small gap between the tank bottom and the printing surface for the next layer to be printed. SLA is used to print materials with a spectrum of different properties including transparent, flexible, heat resistant, castable, and biocompatible pieces [41,42]. SLA also offers very good print resolution (~0.2 µm) at a relatively affordable cost [43]. A new technology utilizing two-photon polymerization was recently introduced to achieve nanometer resolution SLA printing [44]. Although, SLA can be used to produce smooth, high resolution complex architectures, it is still limited to printing a single polymer, by the need for internal supports, and requires post printing cleaning and processing.

### 2.3. Direct Ink Writing (DIW)/Material Jetting

Direct inkjet printing is utilized to deposit materials from inkjet print head onto a build platform or substrate. It depends on the on-demand delivery of adjustable amounts of printable materials onto the printing platform drop-by-drop in a predetermined pattern for layer-by-layer printing. Actuation of material jetting from inkjet head is either thermally or piezoelectrically induced. Thermal jetting requires a heating element that produces a localized heat enough to increase the vapor pressure inside the printing head, leading to ejection of small volume of material. While piezoelectric jetting utilizes a piezoelectric element, which upon application of electric current, generates a mechanical movement enough to eject the ink. An inkjet printing head moving in X, Y and Z directions guides the deposition of a viscous liquid, hydrogel or dispersion onto the printing platform in a desired pattern (Figure 2D) [45]. DIW can be also used to bond powder particles together with the aid of an adhesive polymer [46]. Due to the mechanical ejection mechanism, DIW is time consuming that may take up to a few days for a single print and usually requires post-printing drying. It is utilized for high resolution printing of electronic circuits [47], smooth flexible materials [48], cells and biomaterials [49]. Printing of biomaterials is possible with DIW due to its room temperature piezoelectric printing capabilities and ease of loading into biomimetic printable dispersions.

### 2.4. Selective Laser Sintering (SLS)

Similar to photopolymerization, SLS utilizes a high energy CO_2_ laser beam to melt powder beads into metallic, plastic or ceramic layers. The laser beam scans through the powder bead to print one layer, then printing platform moves down allowing addition of a fresh layer of the powder beads which is then sintered and bind to the previous layer (Figure 2B). This system allows printing complex structures without the need for internal supports, usually required in SLA, while recovered powder after printing can be reused reducing cost and waste [42]. Recently, the use of high energy electronic beam was used to replace laser beam for printing of metallic objects with improved mechanical properties [50]. The use of SLS in sensor fabrication is not common as it is limited to metal and ceramic printing and relatively high operation and maintenance cost compared to other printing techniques.

### 2.5. Tomographic Volumetric Additive Manufacturing

**Figure 3:**
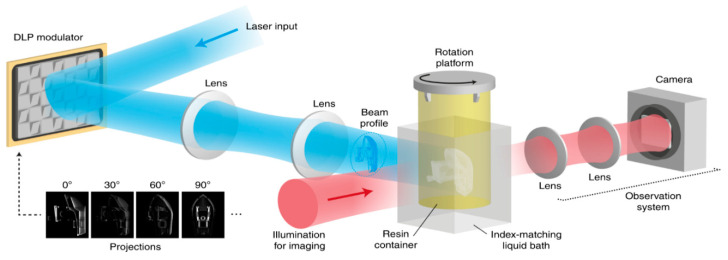
Schematic illustration of tomographic volumetric 3D printing. Reproduced with the permission from [31]. Copyright (2020) Springer Nature available under the terms and conditions of Creative Commons Attribution 4.0 International License.

Unlike other 3D printing techniques that utilize sequential layer-by-layer printing, multi-beam 3D printing technology prints objects by irradiating transparent resin from multiple angles simultaneously (Figure 3). This results in the polymerization of the whole object at the same time, greatly reducing printing time and permitting the production of highly complex architectures [31]. Although it offers a very high throughput (>10^5^ mm^3^/hr), it suffers from low resolution (80 µm) and complex printing setup [51].

### 2.6. Bioprinting

3D bioprinting encompass a spectrum of printing strategies compatible with the labile nature of cells and biomaterials. 3D bioprinting can be adapted in some of the aforementioned 3D printing technologies like direct ink writing, while most of them would have inherent limitations associated with the thermal stability and compatibility of biomaterials. Alternatively, 3D printing techniques, aimed primarily at biomaterial and cell printing, have been developed to overcome these limitations [52]. Syringe-Based extrusion bioprinting, an extrusion-based technique, extrudes a bio-ink at an optimized rate from a moving syringe onto printing platform. Bio-Ink is usually a photocurable polymer or a hydrogel loaded with cells or biomaterials. Extrusion is driven through pneumatic, mechanical, or solenoid valve activation process and the extruded bio-ink is printed layer-by-layer in a computer-aided predetermined pattern. Syringe-Based extrusion is the most widely used bioprinting technique and has been used in most of commercially available bio printers [53]. Syringe-Based extrusion has been mainly utilized in the production of cell-laden architectures for tissue engineering and drug testing [54,55,56]. Another common bioprinting technique is the laser-induced bioprinting, where a biomaterial-laden layer adsorbed on a donor substrate is transferred under the effect of pulsed laser to the receiving substrate. Donor substrate is usually a transparent material like glass coated with laser-absorbing layer that generate a high pressure upon exposure to pulsed laser propelling itself out of the underlying glass onto the receiving substrate [57]. Laser-Induced bioprinting has been investigated for printing of cell-laden collagen architectures and tissue models for cancer studies [58]. Bioprinting promises an easy, accessible, and cost-effective one-step fabrication platform for organs on chip for cancer studies and online high throughput drug-tissue interactions [59,60].

## 3. 3D Printed Electrochemical Sensors

A typical electrochemical biosensor contains two key parts: electrochemical transduction element (e.g., electrode) and biorecognition element (BRE) (e.g., antibody or enzyme). Analyte (e.g., proteins and nucleic acids) from the sample interacts with BRE and generates electroactive products, of which the electrochemical signal is then converted through the transducer and measured. Traditional electrode materials mainly fall into four groups: (1) Noble metals (gold, silver, platinum) have their excellent conductivity, electron transfer kinetics and stability. Gold electrodes are especially favored in bioassays, easy to functionalize with biomolecules and have potential window of about −0.4 to 0.7 V vs. Ag/Ag/Cl at neutral pH [61,62]. (2) Semiconductors, including organic (polymer) and inorganic (indium tin oxide ITO) semiconductors, have lower cost and larger potential window (0.0 to 1.8 V [61]) than gold, but also lower conductivity. (3) Carbon-Based electrodes (pyrolytic graphite, glassy carbon, and graphene) are easy to process, and have large potential window but may have minor stability problems in some applications. (4) Conductive polymers offer a variety of material choices, but have lower conductivity than noble metal and carbon-based electrodes. Electric signals from the electrode are measured by methods such as potentiometry, voltammetry, impedance spectroscopy, conductometry and stripping techniques [63]. Most work discussed in this section employed voltammetry such as cyclic voltammetry (CV) and square wave voltammetry (SWV), and Ag/AgCl as the reference electrode.

Electrodes can be 3D printed using fused deposition modeling (FDM) and selective laser melting (SLM). The printing materials used in FDM are conductive filaments, which are polylactic acid (PLA) or acrylonitrile butadiene styrene (ABS) filament mixed with conductive carbonaceous material such graphite, graphene, carbon nanofibers, carbon nanotubes and carbon black. Two filaments graphene/PLA (Black Magic) and carbon black/PLA (Proto-pasta) are commercially available and can be used in electrode fabrication [64]. SLM uses metal powder to print electrodes, such as iron, steel, and aluminum. 3D printing technique brings great flexibility in electrochemical sensor design. Different electrode geometries can be printed, and the influence on sensor performance studied. The high precision improves the quality of 3D printed sensors, but challenges still exist. FDM printed electrodes have poor conductivity because of the low amount of conductive material in the filament, and therefore need surface treatment before use. Methods such as mechanical polishing, electrochemical or chemical activation and enzyme digestion are used to partially remove the non-conductive material from the electrode surface [64]. SLM 3D printers and metal powders are expensive and require some post-print cleaning [65].

### 3.1. 3D Printed Chip Integrated with Traditional Electrodes

**Figure 4:**
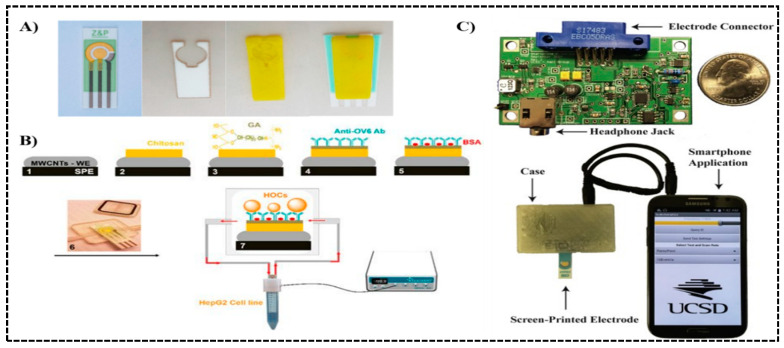
Pre-Fabricated electrodes integrated into 3D printed devices as electrochemical biosensors. (**A**) From left to right: the screen-printed electrode, adhesive layer, 3D printed microfluidic chamber (yellow), and the assembled device. The device was used for detection of liver cancer cell HepG2. Reproduced with permission from [66]. Copyright (2017) Elsevier. (**B**) Immunoassay procedures on a multiwall carbon nanotube (MWCNT) modified screen-printed electrode (SPE) (1–5), electrode-embedded 3D printed flow cell (6), and connected to a flow control system (7), and targeting hepatic oval cells (HOCs). Reproduced with permission from [67]. Copyright (2018) MDPI available under Creative Commons Attribution. (**C**) Printed circuit board module housed in a 3D printed case, with screen-printed electrode inserted, connected to the smart phone for powering, data communication, and display, tracking lung infection in cystic fibrosis. Reproduced with permission from [68]. Copyright (2016) Elsevier.

Here, we class recently reported sensors into two types. The first is traditionally fabricated electrodes integrated into a 3D printed chip. One example is electrochemiluminescent (ECL) sensors, which will be specially covered in next section. Damiati et al. [66] developed such an array for real-time immunodetection of liver cancer cell HepG2. The recombinant S-layer fusion protein (rSbpA/ZZ) was recrystallized on the surface of a screen-printed gold electrode, serving as an intermediate layer to aid the efficient capture of anti-CD133 antibody, which recognizes and binds to the CD133 protein on the surface of liver cancer cells HepG2. A 3D microfluidic chamber was printed by FDM using co-polyester polymer (dimension: 1.5 × 1 × 7 mm) and assembled to the electrode with a double-sided adhesive film (Figure 4A). Cyclic voltammetry (CV) was performed and a detection range of 1 × 10^5^–3 × 10^6^ cells/mL was reported. Similarly, a flow system based on multiwall carbon nanotube (MWCNT) electrode was present by the same group one year later, targeting hepatic oval cells (HOCs), which is an important origin of liver stem cells in hepatocellular carcinoma [67]. The crosslinking chemistry of chitosan and glutaraldehyde was applied on the electrode to immobilize oval cell marker antibody (anti-OV6), which binds to the surface maker OV6 from HOCs (Figure 4B). Digital light processing (DLP) was used to print the flow cell, housing the modified electrode. Square wave voltammetry (SWV) was performed in the assay, and a detection range of 1 × 10^2^–5 × 10^5^ cells/mL was reported. In Sun et al.’s work, the built-in electronics was further expanded to a 1.5 inch × 2.5 inch printed circuit board (PCB) connected to a smartphone, both harvesting energy from the phone and communicating data to the phone for analysis and display [68]. The system was designed to track secretory leukocyte protease inhibitor (SLPI), a biomarker in cystic fibrosis. A case was 3D printed to house the electronics with a screen-printed electrode inserted, then the whole system was connected to a smartphone (Figure 4C). CV was applied and a detection limit of 1 nM was achieved.

**Figure 5:**
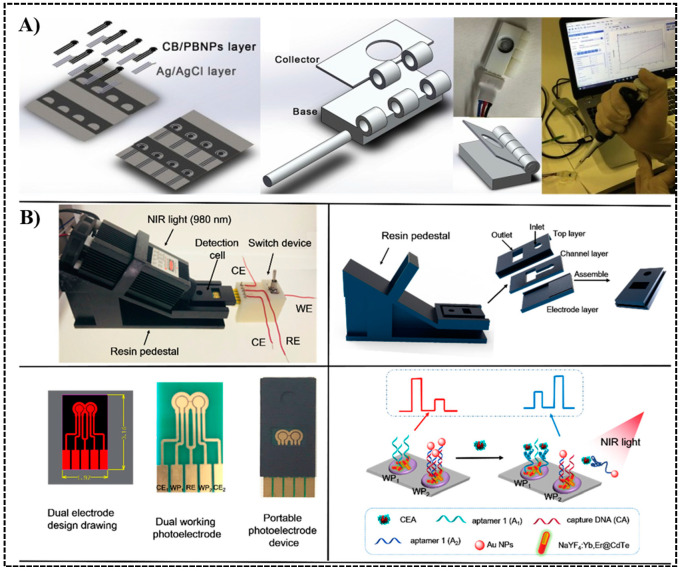
Self-designed electrodes integrated into 3D printed devices as electrochemical biosensors. (**A**) From left to right: wax- & screen-printed paper-based electrode, 3D printed holder for the electrode, and the connected system, used for detection of butyrylcholinesterase activity. Reproduced with permission from [69]. Copyright (2017) Elsevier. (**B**) Dual-Channel ratiometric photoelectrochemical detection of carcinoembryonic antigen (CEA) housed in a 3D printed device. Reproduced with permission from [70]. Copyright (2018) American Chemical Society.

Progress has also been made developing lab-made electrodes, then combining with a 3D printed device. Scordo et al. [69] used wax printing and screen printing to fabricate a paper-based electrode equipped with both reference electrode (Ag/AgCl ink) and working electrode (graphite-based carbon black/prussian blue nanocomposite ink, or CB/PBNBs ink). Preloading the substrate onto the filter paper made this to be an ‘all-in-paper’, ‘reagent-free’ device. A 3D holder was printed by stereolithography (SLA) to house the electrode (Figure 5A). The assay monitored the activity of butyrylcholinesterase using amperometry detection. The sensor achieved a linear range of 1–12 IU/mL with a detection limit of 0.1 IU/mL, and was also tested in serum samples. Tang et al. designed multiple dual (ratiometric) [70,71] and single [72] aptasensors for the detection of carcinoembryonic antigen (CEA), a broad-spectrum biomarker of pancreatic carcinoma, breast cancer and gastric carcinoma, using photo-electrochemistry. The electrode was fabricated from organic or inorganic semiconductors. A 3D printed platform was used to house the whole system (Figure 5B). Photoelectric current was generated by CdTe quantum dots, harvesting light energy from the nanoparticles activated by the near-infrared light. A two working photoelectrode (WP) system was introduced in Figure 5B, where CEA aptamer 1 (A1) was immobilized on WP1, and CEA aptamer 2(A2)-gold nanoparticle conjugate with its complimentary DNA called capture DNA were immobilized on WP2. The binding of CEA to A2 released gold nanoparticles, leading to a signal change between two WPs, from WP1 > WP2 to WP1 < WP2. For detection, the constant potential was set at 0 V and the photocurrent~time curves for both electrodes were recorded. Lowest detection limit achieved in their works was 4.8 pg/mL.

### 3.2. 3D Printed Electrodes

**Figure 6:**
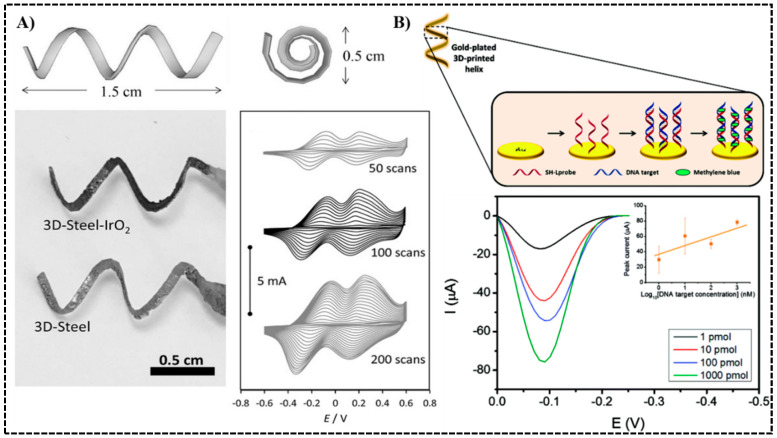
3D printed electrodes as electrochemical biosensors. (**A**) Dimensions of the helical-shaped IrO2-deposited stainless-steel electrode printed by selective laser melting (SLM) and cyclic voltammograms. Reproduced with permission from [73]. Copyright (2015) Wiley. (**B**) Gold-Electroplated helical steel electrode used in measuring DNA hybridization. Differential pulse voltammograms at various DNA concentrations are shown. Reproduced with permission from [28]. Copyright (2017) Royal Society of Chemistry.

Pumera et al. pioneered this area by introducing a helical-shaped stainless-steel electrode made by selective laser melting (SLM) printing [73]. The printed electrode had dimensions of 1.5 cm × 0.5 cm (Figure 6A). After deposition of an IrO_2_ film, the steel-IrO_2_ electrode gave excellent catalytic properties for oxygen generation and as a pH sensor. Moving forward onto biosensors, this group electro-plated the same SLM-printed helical-shaped steel electrode with gold to study DNA hybridization [28]. DNA recognition element SH-L probe was immobilized onto the gold electrode by thiol-gold interactions, the electrode was then blocked to cover any free surface, and incubated with DNA targets for hybridization. Finally, methylene blue solution was added, and methylene blue molecules intercalated into the double helix structure. Differential pulse voltammetry (DPV) was used to measure the reduction peak from the electroactive methylene blue and quantify the extent of DNA hybridization (Figure 6B). A detection range of 1–1000 nM was achieved.

Many researches have detected metals ions and small organic molecules utilizing FDM or SLM printed electrodes, but not much has been done on the biomedical side—some analyte examples are H_2_O_2_, glucose, lactate, and dopamine [64], still not much on proteins and DNA. Several reasons exist: (1) Difficulty in protein/DNA immobilization onto the electrode. (2) Smaller microelectrodes compared to current printed ones are needed for immunoassays. Our group has reported measurements of various cancer biomarker proteins using an eight-electrode array fabricated by inkjet printing using gold nanoparticle ink [48,74,75,76]. Each printed electrode array has an overall surface area of 0.299 ± 0.015 mm^2^, and the electrode contact is only 465 μm × 465 μm (~0.216 mm^2^). The dimensions of reported FDM or SLM printed electrodes are a few cm or larger. (3) High quality electrodes are needed for immunoassays. Interference such as electrode fouling and non-specific binding from the sample matrix are problems for all electrochemical biosensors, but can be a bigger problem in the case of complex biological samples, which puts high requirement on non-specific binding inhibition on the printed electrodes.

## 4. 3D Printed ECL/CL Sensors

Electrochemiluminescent (ECL), chemiluminescent (CL), and nanoparticle-assisted assays utilizing signal amplification strategies have come into the limelight to produce immunosensors that can overcome sensitivity limitations. These immunosensors can have high sensitivity, low detection limits, low background signal, and enhanced signal transduction [77]. ECL biosensors utilize an ECL-active dye as the detection label, responsible for generating the signal via an ECL-producing pathway initiated by a complex redox reaction driven by a conductive electrode interface [78]. ECL active dye emits energy in the form of light as it transits from an excited state produced by the redox chemistry to the ground state when the proper potential is applied. Initial trends involved the development of 3D printed electrodes and channels along with ECL detection. Some of the commonly used ECL substrates include complexes of ruthenium [79,80], iridium [81], and osmium [82]. They can be used in solution, as polymerized films, as nanoparticle bead-based systems or as quantum dots [83,84,85]. Ruthenium poly(vinylpyridine) [Ru(bpy)_2_(PVP)_10_]^2+^ (RuPVP) and ruthenium tris (2,20-bipyridyl) dichlororuthenium-(II) hexahydrate are among the most commonly used substrates [86,87].

ECL assays with 3D printed microfluidic arrays can be automated, cheap, and disposable. Our group used RuBPY silica nanoparticles to evaluate the chemical genotoxicity on DNA damage from cigarettes, electronic cigarettes and in aqueous environmental samples by ECL using a 3D printed device. Here, RuBPY was loaded into silica nanoparticles for signal amplification [88,89]. The redox reaction occurred upon applying a potential of 0.95 V vs SCE to produce ECL at 610 nm with ECL signal proportional to degree of DNA damage. The 3D printed device consisted of three sample chambers running into three detection chambers fitted with a pyrolytic graphite block bearing 10 nm deep nano-wells. The reaction took place in the nano-wells coated with RuBPY/cytochrome P450 enzyme/DNA layers for a layer by layer assembly.

**Figure 7:**
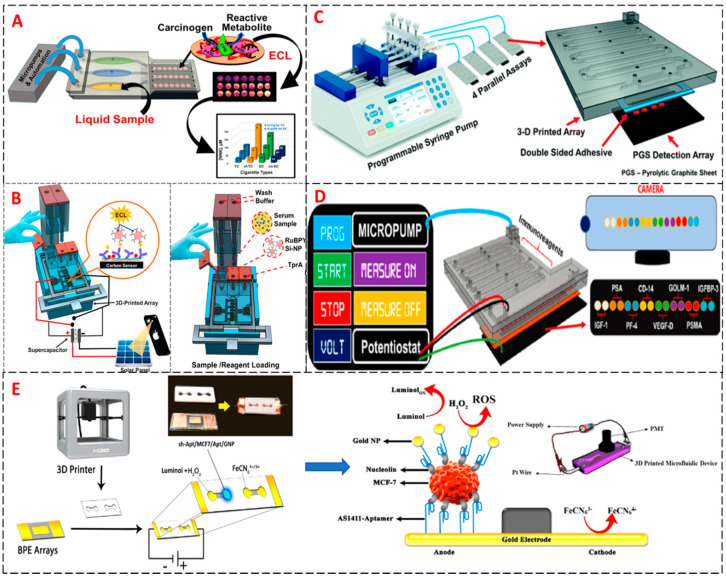
Schematic illustration of 3D printed biosensor arrays that employ electrochemiluminescent (ECL) detection used for cancer diagnostics. (**A**) Automated 3D-printed ECL microfluidic array used in genotoxicity screening Reprinted with permission from Copyright (2017) American Chemical Society. (**B**) Automated 3D printed supercapacitor-powered ECL Protein Immunoarray. Reproduced with permission from [90]. Copyright (2016) Elsevier. (**C**) Automated 3D printed microfluidics immunoassay detecting 4 protein samples simultaneously. Reproduced with permission from Copyright (2018) The Royal Society of Chemistry. (**D**) Automated 3D printed microfluidic array for detection of 8 cancer biomarker proteins simultaneously. Reproduced with permission from [90]. Copyright (2018) American Chemical Society. (**E**) Complete pathway to the detection of human breast cancer cells by using bipolar electrode modified by an aptamer coated inside a 3D printed microchannel by ECL. Reproduced with permission from [92] Copyright (2018) Elsevier.

We also developed 3D printed immunoarrays automated by a programmable syringe pump delivering reagents sequentially into the detection chambers. The sandwich immunoassay was carried out at the detection chamber of 10 nm deep nano-wells on a pyrolytic graphite chip, facilitating lower sample and reagent volumes. The size of the sample chamber was governed by the number of cancer biomarker proteins being detected, starting with 3 biomarkers [90] and moving up to detect 8 biomarkers simultaneously in a single array [91]. In all the devices, the detection chamber was designed to accommodate a working and reference electrode to which potential was applied to generate ECL light in the presence of the co-reactant triproplyamine (TPrA). Images of the ECL signal intensity were captured in a dark box with a CCD (charged couple device) camera (Figure 7). In an early experiment, our group used a non-transparent 3D device printed by FDM 3D printer that had a sample chamber and reagent reservoirs to facilitate gravity driven reagent delivery. The system was powered by a capacitor without a potentiostat (Figure 7B). Electrodes were screen printed and then functionalized with capture antibodies. RuBPY SiNPs coated with secondary antibodies were used to carry out the immunoassay on the surface of the electrode. 3 prostate cancer biomarkers were measured, prostate-specific antigen (PSA), prostate-specific membrane antigen (PSMA) and platelet factor 4 (PF4). Limits of detection (LODs) ranged from 300–500 fg/mL [91]. After this initial system, designs evolved to include many improvements. The 3D printed devices moved to semi-transparent devices, SLA printers were used for array printing, Krylon spray was used in order to make the devices more transparent, pyrolytic graphite blocks were replaced by thin pyrolytic graphite sheets, the cost of development decreased. Finally, panels of biomarkers were expanded for more reliable prognosis and treatment [90,92]

In related work, Montaghi et al. developed a system for sensitive detection of breast cancer cells (MCF-7) using ECL via a functionalized bipolar electrode (BPE) mounted in a 3D printed microchannel (Figure 7E) [93]. Functionalization involved attachment of aptamer specific to nucleolin on the anode of the BPE. Gold nanoparticles were modified by a secondary aptamer. ECL was generated using luminol in the presence of hydrogen peroxide. The assay was able to detect limit of 10 breast cancer cells MCF-7.

Chemiluminescence (CL) can also be used to determine the concentration of an analyte by measurement of the luminescence intensity initiated by a chemical reaction [94]. Unlike ECL, CL has no need for electrodes and requires on light detection for operation [95,96]. The most commonly used CL substrates are luminol with peroxidases and alkaline phosphatase (ALP) for activation. The signal is generated from the reaction between the CL substrate and hydrogen peroxide in the presence of horseradish peroxidase enzyme (HRP) as the catalyst, emitting light at 425 nm when the oxidized triplet dianion decays from its excited state to the ground state. This system has wide applications to immunoassays, environmental analysis [97,98], clinical diagnosis [99,100], the food safety [101,102], and pharmaceutical analysis [103,104]. Gold nanoparticles (AuNPs) have been used to label antibodies and enhance CL signals [105]. Similarly, polymers multi-labelled with enzymes like poly-HRP has been recently investigated to enhance the CL signal increasing assay sensitivities [106]. Signals are measured in a dark box using a CCD camera same as in ECL detection. CL combined with 3D printing technology opened doors to automation and multiplexed detection of cancer biomarker proteins [107,108].

**Figure 8:**
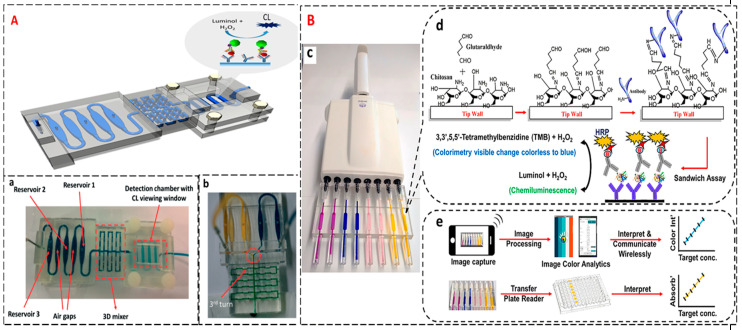
Schematic illustration of 3D printed biosensor arrays that employ chemiluminescent (CL) detection for cancer diagnostics. (**A**) 3D printed design of a unibody microfluidic CL array device. Inset: (**a**) Details of the unibody immunoarray showing upstream reservoir chambers separated by air chambers for air gaps to prevent intermixing, followed by a 3D mixing network of 96 turns, finally detection chamber that houses the antibody array; (**b**) The mixer highlight containing 96 turns that are 0.8 mm × 0.8 mm × 0.8 mm 90 turns. 2 different solutions pumped into the mixer at the rate of 50 μL/ min mix at the third turn (indicated by the arrow). It shows excellent mixing efficiency (indicated by the difference in the colors before and after mixing). Reproduced with permission from [109]. Copyright (2017) The Royal Chemical Society. (**B**) Graphical representation of ELISA sandwich immunoassay in 3D printed pipette tips. Inset: (**c**) Fully transparent 3D printed pipette tips filled with different color food dyes attached to a multi tip pipette; (**d**) steps involved in the pre coating showing the immobilization of capture antibodies on the inner walls of the tips coated with chitosan followed by the sandwich immunoassay and the generation of the CL signal and colorimetry; (**e**) Signal capture and processing flow for both colorimetry and CL using a smartphone and a microplate reader. Reproduced with permission from [29]. Copyright (2019) American Chemical Society.

We reported the first non-polydimethylsiloxane (PDMS), transparent 3D printed device with channels, detection chamber and reagent mixer (Figure 8A) integrated with an immunoarray in 2017 to measure protein biomarkers. Proteins PF-4 and PSA were studied in this example, giving LODs of 0.5 pg/mL for both along with broad dynamic ranges [109]. We also developed an assay using CL for multiplexed ELISA in 3D printed pipette tips (Figure 8B) [29]. Both colorimetric and chemiluminescence detection methods were considered in the novel TIP ELISA approach. A smartphone was utilized to enable the electronic delivery of results proving it to be suitable for POC testing. The immunoassay was done in the side of the pipette tips. It proved to be more sensitive, faster and required less sample and reagent volumes than traditional ELISA assays. Four prostate cancer biomarkers were studied giving LODs down to 0.5 pg/mL concentration level. The results showed good correlations with ELISA cutting down the cost to less than 25% of conventional ELISA. Other 3D printed systems that have been developed that detect lactate and H_2_O_2_ in biological fluids and plant extracts.

## 5. Hybrid 3D Printed Sensors

Hybrid sensors with capability to integrate multiple components play a crucial role in developing newer technologies and deliver better user interaction. Ability to design and fabricate such integrated hybrid sensors during the proof of concept stage provides greater advantage than using simple off the shelf commercial sensors. Such an attempt requires addressing unique challenges, and 3D printing with ability to make complex shapes and sizes, using multi-material, nano-material integration and 3 dimensional structures using conductive inks/materials will provide enhanced sensing capabilities. Most importantly, 3D printing aids in consolidating a working prototype of such hybrid sensors with less creation time and cost.

Integration of 3D printed electrodes made of materials composed of metal-based inks and conductive materials have played a key role in development of novel electrochemical and electro-optical micro devices and they offer several benefits 1. Rapid prototyping—full realization of a manufactured prototype can be improved 2. Manufacturing tailor made electrodes and seamless incorporation—ideal for designing miniaturized point-of-care devices 3. Ability to design features not possible by traditional methods like rough/smooth surfaces, multiple electrodes for multiplexing, control of sensor sizes, complex geometry, cost to prototype, robustness and chemical resistance supported my availability of novel nano composite materials. Evolution of electrochemical 3D printed sensors predominantly focused on making 3D printed housing to integrate commercially available electrodes. Thus, making a functional hybrid sensor that has all the components printed and integrated will help realize the novel technologies reach the commercial arena at a scale approachable by masses. Biomarker discovery and cancer diagnostics are particularly challenged by lack of standalone devices that are sensitive to detect ultra-low levels of the biomarker levels with multiplexing capabilities. 3D printing can facilitate integration of multiple components like reagent storage and delivery, sensing surfaces by printing bio-recognition surfaces, complementary electronics for automation and data sharing modules in a miniaturized format realizing a true point of need platform.

**Figure 9:**
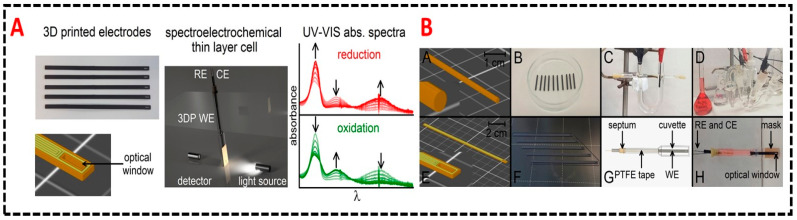
(**A**) Schematic illustration of 3D printed software designs and scheme of hybrid sensors arrangement with an optical window on electrode surface in the path of light beam. A complete UV-Vis spectrum with all the absorption transients shown as inset for successful description of integration. (**B**) Images of the working set up and pictures of the 3D printed electrodes and quartz housing for UV-Vis measurements. All components arrangement, working- counter—and reference electrode arrangement along with optical viewing window shown. Reproduced with permission from [110]. Copyright (2019) Elsevier.

We summarized such recent hybrid systems here, Sebechlebska et al. demonstrated a 3D printed hybrid integrated sensor that integrates electrochemistry and UV/Vis absorption spectroscopy, dubbed as a first ever report of UV/Vis absorption spectroelectrochemical apparatus, employing 3D printed optically transparent working electrodes [110]. PLA based 3D printed electrodes made from carbon nanotubes (Figure 9A) were utilized in this study due to their higher electrical conductivity compared to PLA doped with carbon black and graphene. Functional electrode sensors require facile electron transfer at electrode/electrolyte interface, variation in electroactive probes reversibly transferring electrons at electrode surface is referred to intrinsic kinetic barrier. Majority of PLA/carbon composited have high kinetic barrier not suitable for electrochemical studies, whereas Ruthenium (III) acetylacetonate based activation process evolved a lowest ever reported kinetic barrier for a 3D printed electrode with magnitude of faradaic response like conventional carbon electrode (Figure 9A). A 3-electrode (gold wires as counter and reference electrodes with PLA/CNT electrodes as working electrodes) setup integrated in a Quartz cuvette was used to monitor electrochemical process at 3D printed electrode by in-situ UV/Vis absorption spectroscopy. PLA/CNT electrode was designed with optical window at the bottom end to accurately visualize UV-Vis spectra of electrochemically active species (Figure 9B). UV-Vis spectra obtained by cyclic voltammetry of Ru(acac)3 showed reduction and subsequent re-oxidation of the electroactive species. The rate of absorbance change in reduction step decreases with time confirms depletion of reactant at working electrode optical window. All the absorption transients suggested successful implementation of a hybrid sensor using 3D printed nanocomposite-based electrodes.

**Figure 10:**
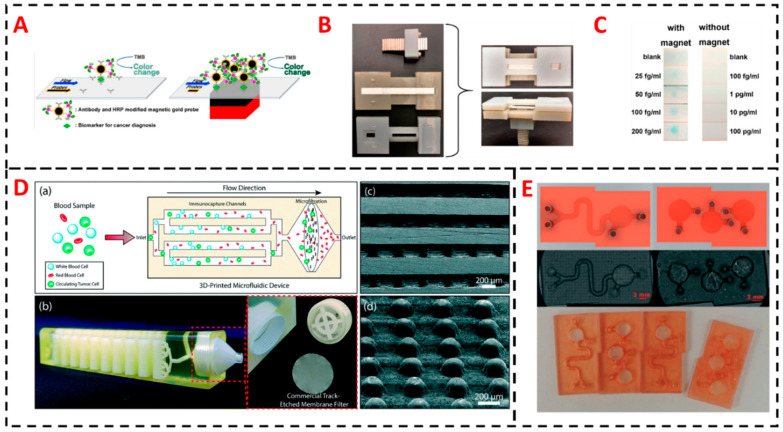
(**A**) Schematic representation of effect of magnetic focusing on a lateral flow assay resulting in enhanced density of magnetic probe-labelled target in the capture antibody detection zones. Effect of magnetic focus represented with and without magnets underneath lateral flow (LF) device to show improved accumulation compared to conventional lateral flow assay (LFA). (**B**) 3D printed device that acts like a frame to hold the lateral flow strip along with magnet and a sample addition zone for liquid biopsy sample aimed to detect cancer biomarkers. (**C**) Comparison of colorimetric signal as detection results with and without magnet. Reproduced with permission from [111]. Copyright (2019) American Chemical Society (**D**) Schematics of tumor cells enrichment process in a multi-layered immunocapture microfluidic layer. Microposts inside the microfluidic channels assist structural integrity and enhanced surface area to allow higher capture and enrichment efficiency. 3 µM membrane filter to retain all eluted nucleated cells for downstream applications. Reproduced with permission from [112]. Copyright (2019) The Royal Society of Chemistry (**E**) Images of 3D printed modular chips made from DLP based stereolithography based approach where monomeric resin is doped with acrylic acid to generate a platform that has intrinsic carboxylates for direct conjugation of biomolecules. Reproduced with permission from [113]. Copyright (2019) The Royal Society of Chemistry.

In another attempt to integrate unique methodologies to produce a hybrid 3D printed sensors, Irudayaraj et al. integrated magnetic field/magnetic focus with lateral flow sensor to increase residence time of target-ligand interaction in turn resulting in enhanced sensitivity compared to conventional lateral flow immunoassay (LIFA) for liquid biopsy and tissue samples (Figure 10A). The proposed magnetic focus lateral flow sensor (mLFS) [111] was implemented in detection of cervical cancer biomarker valosin-containing protein (VCP) as proof of concept with exceptional detection limits of 25 fg/mL with enhanced sensitivity of 10^6^ fold improvement over conventional lateral flow assays. Magnetic focus is provided by a controlling a simple magnet to manipulate magnetic probe-labelled targets with capture antibodies at the detection zones. Slower the movement the higher the interaction and higher the number of labelled probe targets at detection zone. A simple setup of 3D printed frame designed to integrate lateral flow strip with magnetic bar along with the sample application portal allowed simple operations process (Figure 10B). Thorough analysis for contribution of improved sensitivity by magnetic focus was demonstrated by surface-enhanced Raman spectroscopy (SERS) and dark field imaging of the magnetic nanoparticles along with particle image velocimetry. Signal generation on the lateral flow assay was via color change in presence of 3,3′,5,5′-Tetramethylbenzidine (TMB) substrate, magnetic nanoparticle probes modified with HRP and antibody resulted in generation of color spots at detection zones upon addition of colorimetric substrate (Figure 10C).

Sarioglu et al. [112] constructed a hybrid 3D printed monolithic device for negative enrichment of circulating tumor cells from whole blood, by combining microfluidic immunoaffinity based cell capture and a membrane filter to enrich the captured circulating tumor cells for downstream applications. Microfluidic device was designed to have high surface area and increased interaction between white blood cells and functionalized surfaces with 4-32 stacked microfluidic layers and 200 µM diameter microposts (Figure 10D). The microposts serve as support of microfluidic layers as well as chemically functionalized to attach neutravidin for CTC’s immunoaffinity capture. Filtration section of the platform has track etched membrane filter to facilitate minimizing cell loss from sample during enrichment and to collect capture CTCs from the device for downstream application like fluorescence microscopy or on-chip staining. Blood sample premixed with biotinylated anti-CD45 antibody to pass through Neutravidin functionalized stacked microfluidic layers. Typically, ~90% of tumor cell recovery was found with multiple cancer cell lines with ability to process clinically relevant blood volumes with at least 300 µL per microfluidic layer.

Frascella et al. demonstrated a hybrid sensor that possess active functional groups to conjugate biomolecules, they designed a photocurable formulation to introduction desired amount of carboxyl (-COOH) groups [113]. 3D printing with intrinsic functionalities, especially in terms of ability to easily immobilize biomolecules on polymeric surfaces is very attractive mainly because it reduces complexity of sensor manufacturing, no multiple components needed to make a functional platform and most importantly in a mass production scenario it allows reproducibility. Frascella et al. designed simple 3D printed sensors with Y-shaped mixer for single step reagent mixing assays and multiple step single chamber devices (Figure 10E) that require no chemical derivatization and showed application of colorimetry detection of cancer biomarker proteins. Three formulations of chemically modified acrylic resin bisphenol A ethoxylate diacrylate (BEDA), 1,6-hexenediol diacrylate (HDDA) and poly(ethylene glycol) diacrylate (PEGDA) were evaluated for their protein grafting ability post 3D printing and found out BEDA at 10% acrylic acid density showed highest amount of protein immobilization and subsequent detection via colorimetric signal generated by HRP labelled antibodies. Two angiogenesis biomarkers, vascular endothelial growth factor and angiopoietin-2 were detected in serum with detection limits of 11 ng/mL and 0.8 ng/mL, respectively.

Above mentioned proof-of-concept platforms highlights the need for hybrid sensors with intrinsic capabilities to evolve into true fully 3D printed diagnostic platforms. Overall, 3D printing of biosensors for diagnostics is rapidly evolving and moving in a direction to address many inherent challenges. By designing hybrid sensors with ability to integrate multiple technologies and improving biocompatibility and bio-adhering capabilities is establishing new benchmarks and a path to transform from just prototyping to commercial mass production market.

## 6. Challenges and Future Perspectives

Assay systems described above demonstrate that 3D printing has offered a great new low cost, asset for researchers developing biosensors. A major advantage is that sensor arrays for proteins and other analytes can be developed and fabricated on the same platform, the final optimized prototype can essentially be the final product for the clinic or hospital. That along with the speed of development and optimization, and the low cost of most of these printers, suggest a brilliant future for 3D-printed diagnostic devices measuring biomarkers of all types for cancers and other diseases. Gained protein biomarker multiplexing capabilities, increasingly supported by 3D printed immunosensors, is of great interest specially with the growing knowledge correlating different abnormalities in biomarker expression with different types of cancer. Table 1 summarize examples of protein biomarkers detection strategies and its correlated cancer.

One radical foreseen change that can be realized via 3D printing, is the fabrication of sensors with integrated bio-recognition elements. Most conventional sensor assembly strategies require extensive procedures to decorate the sensor interface with biomolecules to selectively capture target analytes. This is an area where 3D printing can stand out as an approach to overcome tedious interface biomolecule decoration steps. 3D printing of biomolecules is gaining great progress in this direction specially with the rapid development in 3D bioprinting where enzymes, proteins and cells can be directly integrated in the printed matrix. Some 3D printed biosensors have been printed with integrated biomolecules [114] and different printing strategies are being extensively directed towards this goal, like syringe-based or laser-induced bioprinting.

In spite of these advantages, 3D printing is still limited in aspects of multi-material printing and resolution. Developing 3D printed electrochemical sensors for cancer protein biomarkers usually necessitates multicomponent to be printed which is hindered by the very limited printing techniques capable of achieving such feature and the high variation in inter-material adhesion forces. By way of example, incorporating a conductive electrode material into a 3D printed sensor using fused deposition modeling require using conductive carbonaceous filaments that can be printed onto conventional nonconductive filaments. Although achievable [37], this process is quite complex and require extensive optimization of the printing parameters to avoid leakage and/or structure deformity. In addition, common 3D printing techniques and materials have limited compatibility with biomolecules especially with high energy required for printing processes, like high heat in FDM and high energy laser in stereolithographic 3D printing, that prevent direct printing of these biomolecules. This necessitates a post printing surface modification steps to improve the surface characters or add functionality where biorecognition moieties could be immobilized.

These drawbacks are driving research boundaries of 3D printing to address new challenges and prior limitations. Recently, 3D printing has been utilized for in situ printing of deformable sensors right onto soft tissues and organs to accommodate its movement and expansions [115]. This flexibility in the applications of 3D printing materials with different characteristics expose the power of this technique in exploring what has been previously limited to sophisticated equipment and complex fabrication facilities. Ability to integrate complex architectures in a multicomponent sensor, in one step, is a crucial progress that reduce sensor production and testing time, bring more sophisticated sensor designs, and allow the production of sensor at the point of need. 3D printing is also transforming from the method of choice for prototyping to a high scale production technique, due to progressive availability of high throughput desktop 3D printers with orders of magnitude larger printing surface compared to earlier printers. Sensors availability and affordability, granted by current 3D printing ventures, may help diagnose patients at very early stage where the disease is most responsive to treatment. 3D printing sensors with integrated biomaterials and signal readout through simple connection to portable devices like mobile phones, may shape the future of POC

## Figures and Tables

**Table 1:** A summary of published biomarker-based cancer diagnostics.

Cancer	Biomarker	Sensor	Detection Range or Limit
**Liver cancer**	CD133	Screen-printed gold electrode integrated into a 3D printed chamber	1 × 10^5^–3 × 10^6^ HepG2 liver cancer cells/mL [66]
**Hepatocellular carcinoma**	Oval cell marker antibody (OV6)	Multiwall carbon nanotube (MWCNT) functionalized electrode integrated into a 3D printed flow cell	1 × 10^2^–5 × 10^5^ hepatic oval cells (HOCs)/mL [67]
**Cystic fibrosis**	Secretory leukocyte protease inhibitor (SLPI)	Printed circuit board with built-in screen-printed electrode integrated into a 3D printed case and connected to a smart phone for control	Limit of 1 nM [68]
**Pancreatic carcinoma, breast cancer and gastric carcinoma**	carcinoembryonic antigen (CEA)	Self-designed and printed photoelectrode integrated into a 3D printed platform	10.0 pg/mL–5.0 ng/mL with limit of 4.8 pg/mL [70]
**Prostate cancer**	Prostate-Specific antigen (PSA), prostate-specific membrane antigen (PSMA)	3D printed multiplexed ECL immunoarray with programmable syringe pump	Limits of 150 fg/mL for PSA, and 230 fg /mL for PSMA [92]
**Prostate cancer**	PSA, cluster of differentiation 14 (CD-14), Golgi membrane protein 1 (GOLM-1), insulin-like growth factor binding protein 3 (IGFBP-3), insulin-like growth factor 1 (IGF-1), platelet factor 4 (PF-4), vascular endothelial growth factor D(VEGF-D), PSMA	3D printed multiplexed ECL immunoarray with lab-built electronic control system	Limits of 78−110 fg /mL [90]
**Prostate cancer**	PSA, PSMA, PF-4	3D printed multiplexed ECL immunoarray powered by supercapacitor	Limits of 300–500 fg/mL [91]
**Breast cancer**	Nucleolin	Functionalized bipolar electrode (BPE) mounted in a 3D printed microchannel for ECL detection	Limit of 10 MCF-7 breast cancer cells [93]
**Prostate cancer**	PSA, PS-4	Unibody 3D printed multiplexed CL immunoarray	Limits of 0.5 pg/mL [109]
**Prostate cancer**	PSA, VEGF, IGF-1, CD-14	ELISA based 3D printed multiplexed pipette tip for CL and colorimetric detection	Limits of 5 pg/mL for PSA, 25 pg/mL for VEGF, 2.5 pg/mL for IGF-1, and 0.5 pg/mL for CD-14 [29]
**Cervical cancer**	Valosin-Containing protein (VCP)	Magnetic focus lateral flow immunosensor (mLFS) integrated into a 3D printed frame for colorimetric detection	Limit of 25 fg/mL [111]
**Ovarian cancer, breast cancer**	VEGF, angiopoietin-2 (Ang-2)	3D printed immunoarray using lab-formulated carboxyl group rich resin for colorimetric detection	Limit of 11 ng/mL for VEGF, and 0.8 ng/mL for Ang-2 [113]
